# TCR Triggering by pMHC Ligands Tethered on Surfaces via Poly(Ethylene Glycol) Depends on Polymer Length

**DOI:** 10.1371/journal.pone.0112292

**Published:** 2014-11-10

**Authors:** Zhengyu Ma, David N. LeBard, Sharon M. Loverde, Kim A. Sharp, Michael L. Klein, Dennis E. Discher, Terri H. Finkel

**Affiliations:** 1 Department of Biomedical Research, Nemours/A.I. duPont Hospital for Children, Wilmington, Delaware, United States of America; 2 Department of Chemistry, Yeshiva University, New York, New York, United States of America; 3 Department of Chemistry, College of Staten Island, City University of New York, Staten Island, New York, United States of America; 4 Department of Biochemistry and Biophysics, University of Pennsylvania, Philadelphia, Pennsylvania, United States of America; 5 Institute for Computational Molecular Science and Department of Chemistry, Temple University, Philadelphia, Pennsylvania, United States of America; 6 Department of Chemical and Biomolecular Engineering, University of Pennsylvania, Philadelphia, Pennsylvania, United States of America; 7 Department of Pediatrics, Nemours Children’s Hospital, Orlando, Florida, United States of America; 8 Department of Biomedical Sciences, University of Central Florida College of Medicine, Orlando, Florida, United States of America; J. Heyrovsky Institute of Physical Chemistry, Czech Republic

## Abstract

Antigen recognition by T cells relies on the interaction between T cell receptor (TCR) and peptide-major histocompatibility complex (pMHC) at the interface between the T cell and the antigen presenting cell (APC). The pMHC-TCR interaction is two-dimensional (2D), in that both the ligand and receptor are membrane-anchored and their movement is limited to 2D diffusion. The 2D nature of the interaction is critical for the ability of pMHC ligands to trigger TCR. The exact properties of the 2D pMHC-TCR interaction that enable TCR triggering, however, are not fully understood. Here, we altered the 2D pMHC-TCR interaction by tethering pMHC ligands to a rigid plastic surface with flexible poly(ethylene glycol) (PEG) polymers of different lengths, thereby gradually increasing the ligands’ range of motion in the third dimension. We found that pMHC ligands tethered by PEG linkers with long contour length were capable of activating T cells. Shorter PEG linkers, however, triggered TCR more efficiently. Molecular dynamics simulation suggested that shorter PEGs exhibit faster TCR binding on-rates and off-rates. Our findings indicate that TCR signaling can be triggered by surface-tethered pMHC ligands within a defined 3D range of motion, and that fast binding rates lead to higher TCR triggering efficiency. These observations are consistent with a model of TCR triggering that incorporates the dynamic interaction between T cell and antigen-presenting cell.

## Introduction

T cells recognize antigens through the binding between T cell receptors (TCRs) and peptide-major histocompatibility complexes (pMHCs) at the interface between T cell and antigen presenting cells (APCs). pMHC-TCR binding triggers TCR signaling that activates T cells. T cell activation initiates T cell-mediated adaptive immune responses, which are responsible for pathogen clearance or autoimmune disease, depending on the source of peptide antigen. Despite its critical importance, it remains unclear how specific pMHC-TCR binding initiates, or triggers, a signal from the TCR in the first place. The mechanism of TCR signal initiation, also called “the TCR triggering puzzle”, cannot be explained by classical models such as receptor conformational change or crosslinking [1], [2].

A key feature of TCR triggering is the two dimensional (2D) nature of pMHC-TCR interaction. pMHC and TCR are anchored on plasma membranes and their movement is limited to 2D diffusion. The binding between pMHC and TCR, therefore, can only occur when the two plasma membranes are brought together through cell-cell contact and are closely aligned by adhesion molecules. The membrane-membrane contact, however, is not static. The T cell-APC interaction is dynamic and their relative motion inevitably applies mechanical stress to the interacting membranes and pMHC-TCR binding. Several models of TCR triggering have been proposed by taking into consideration certain features of the complex 2D pMHC-TCR interaction. The kinetic segregation model of TCR triggering, for example, proposes that the closely aligned membranes create steric barriers that segregate surface molecules based on their size [3]. The exclusion of large molecules such as tyrosine phosphatase CD45 from the vicinity of bound pMHC and TCR, which are both relatively small, initiates TCR signaling by creating tyrosine kinase-rich zones around the TCR. The receptor deformation model, on the other hand, postulates that the binding between membrane-anchored pMHC and TCR transfers mechanical forces associated with cell locomotion to the TCR/CD3 complex. The mechanical forces deform the TCR/CD3 into a conformation or configuration that favors signal initiation [4], [5].

The binding properties that determine the efficiency of TCR triggering should also be considered in a 2D context. The kinetics of 3D binding is largely determined by the overall binding activation energy and bond formation detail at the binding interface. 2D binding kinetics, on the other hand, is additionally influenced by factors such as ligand and receptor size, lateral diffusion rate, pre-aligned binding interface [6], [7], and mechanical stress associated with membrane dynamics [8]. Studies on the relationship between the 2D kinetics of pMHC-TCR binding (on-rate, off-rate and affinity) and its signaling potential have started to emerge recently [9], [10], [11]. The results, however, have been highly inconsistent.

To delineate how the 2D nature of pMHC-TCR interaction contributes to TCR triggering, here we altered the 2D pMHC-TCR interaction by tethering pMHC on surfaces with flexible poly(ethylene glycol) (PEG) polymer linkers of varying lengths, and compared their effects on T cell activation. With increase in polymer length, tethered pMHC ligands have an increased range of motion in the third dimension. Thus, the pMHC-TCR interaction becomes more 3D-like. We found that pMHC ligands tethered with PEG polymers of up to 380 nm were capable of triggering TCR. The efficiency of triggering, however, gradually decreased with increase in linker length. Molecular dynamics simulation suggested that pMHC tethered with longer polymers binds its receptors with slower on-rates and off-rates. These observations are consistent with the receptor deformation model of TCR triggering.

## Results

### Tethering pMHC Ligands to a Surface with PEG Polymer Linkers

**Figure 1:**
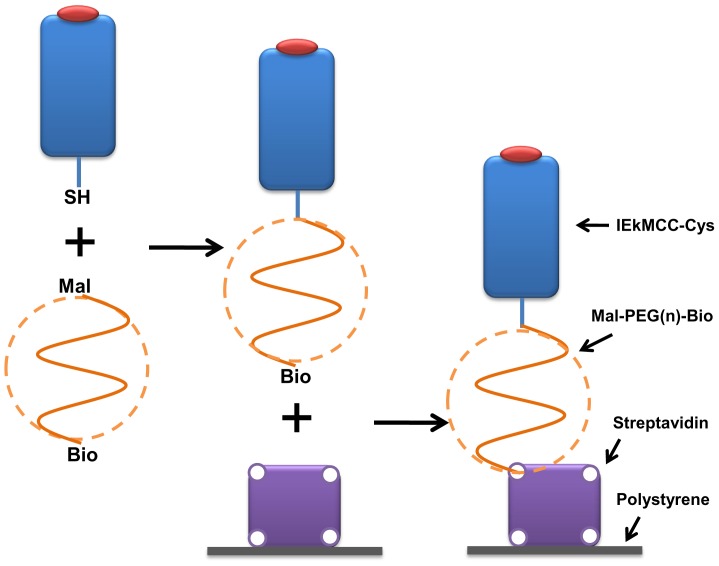
Schematic illustration of IEkMCC ligands tethered onto a plastic surface with PEG polymer linkers. IEkMCC proteins with free c-terminal cysteines were first conjugated with heterobifunctional PEG linkers Mal-PEG-Bio through interactions between the sulfhydryl group and the maleimide group. Conjugates with biotin at the free ends of the polymer were then tethered to a plastic surface coated with streptavidin.

**Figure 2:**
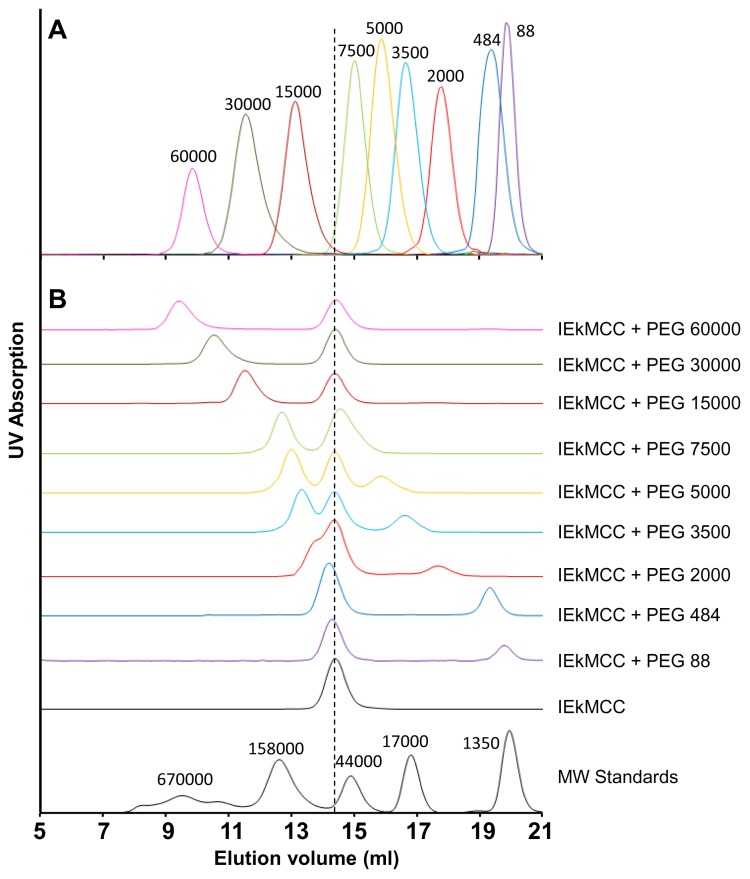
Characterization and separation of PEG polymer linkers and IEkMCC-PEG conjugates. (A) Compiled elution curves of nine PEG polymer linkers from a Superdex 200 10/300 GL gel filtration column. The polymers were detected through the weak UV absorption of the biotin group using a 245 nm UV detector. (B) Separation of the IEkMCC and PEG polymer reaction products. The reaction products were loaded on a Superdex 200 10/300 GL gel filtration column to separate IEkMCC-PEG conjugates, unreacted IEkMCC, and unreacted PEG polymers. The reaction products of PEG 15000, PEG 30000 and PEG 60000 were first purified with an IEk-binding affinity column to eliminate unreacted PEG polymers. The dotted vertical line indicates the elution volume of IEkMCC protein. The late elution peaks of unreacted polymers can be seen for PEGs ranging from PEG 88 to PEG 5000. In reaction with PEG 7500, unreacted IEkMCC and unreacted polymer formed a single peak that was eluted at a position between unconjugated IEkMCC and pure PEG 7500.

To tether pMHC ligands to a surface using PEG linkers, we first conjugated pMHC with a PEG polymer, then tethered the pMHC-PEG conjugates onto a plastic surface through biotin-streptavidin interactions (Fig. 1). To this end, mouse MHC class II molecule IEk with covalently linked moth cytochrome c (MCC) peptide (aa88-103) was engineered to have a free cysteine at the C-terminal end of the IEk β chain. The protein was expressed in a baculovirus insect expression system in secreted form and purified with affinity chromatography (Fig. S1A) [12], [13]. The purified protein eluted as 50 kDa monomers in gel filtration chromatography (Fig. S1B). The protein was then conjugated with hetero-bifunctional polymer linker Maleimide-PEG-Biotin (Mal-PEG-Bio) through the reaction between the maleimide group of the PEG linker and the sulfhydryl group (−SH) of the protein C-terminal cysteine. Nine PEG linkers of different lengths with molecular weights ranging from 88 to 60000 Da were used (Table 1). In gel filtration, these polymers were eluted in the expected volumes and order (Fig. 2A). After the reaction, the mixture containing IEkMCC-PEG conjugates, unreacted IEkMCC, and unreacted PEG polymers was subjected to chromatography for separation (Fig. 2B). The three products of the reaction with mid-length polymers (PEG 3500, PEG 5000, and PEG 7500) were separated by a single round of gel filtration chromatography and the conjugate peaks were collected. For reactions with large polymers (PEG 15000, PEG 30000, and PEG 60000), the conjugates and the polymers could not be separated by gel filtration. The polymers were therefore first eliminated with an IEk-specific antibody 14-4-4s affinity column. The remaining IEkMCC-PEG conjugates and free IEkMCC were then separated using gel filtration. For reactions with small polymers (PEG 88, PEG 484 and PEG 2000), gel filtration could not separate IEkMCC and conjugates, but polymers could be eliminated. In this case, the peaks containing both IEkMCC and conjugates were collected. The concentrations of those conjugates were calculated based on measured biotin concentration and the knowledge that each IEkMCC molecule can have at most one biotin. Any free IEkMCC in the mixture has no affect on the subsequent tethering step since it cannot bind streptavidin.

**Table 1:** PEG linkers and properties.

Linkers	MW (Dalton)	Number of PEO units	Contour length (nm)^1^	Flory radius (nm)^2^	FRET efficiency (%)
**PEG 88**	88	2	0.6	0.5	58
**PEG 484**	484	11	3.1	1.2	53
**PEG 2000**	2000	45	12.7	2.8	34
**PEG 3500**	3500	80	22.3	3.9	20
**PEG 5000**	5000	114	31.8	4.8	14
**PEG 7500**	7500	170	47.7	6.1	2
**PEG 15000**	15000	341	95.5	9.3	–
**PEG 30000**	30000	682	190.9	14.0	–
**PEG 60000**	60000	1364	381.8	21.3	–

1 PEG contour length is calculated based on the PEO unit length of 0.28 nm in water [32].

2 The Flory radius (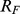) of the PEG polymer of 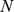 subunits and unit length 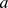 was calculated using 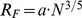, where 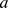 is 0.28 nm.

As shown with the gel filtration analyses (Fig. 2A), the hydrodynamic sizes of the polymers are much larger than those of globular proteins of similar molecular weights, indicating a relatively extended conformation of the PEG polymers in aqueous solution. For example, PEG 7500 was eluted with a similar volume to a protein with molecular weight of 44000 Da. Conjugation of PEG polymers significantly increased the hydrodynamic size of IEkMCC protein (Fig. 2B). Addition of a PEG 7500 polymer to IEkMCC almost doubled its apparent molecular weight, leading to baseline separation of the conjugates and unreacted IEkMCC. Consistent with their monomeric nature, the conjugates did not activate T cells when used in solution even at high concentrations (data not shown).

### FRET Characterization of Surface-tethered pMHCs

**Figure 3:**
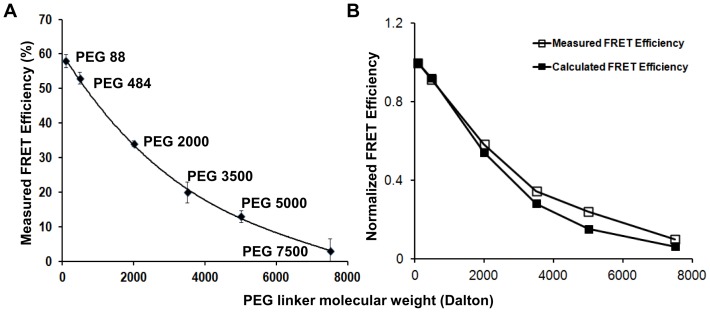
FRET between streptavidin on plastic plates and IEkMCC tethered with PEG polymers. (A) Measured FRET efficiencies of IEkMCC tethered with six different PEG polymers. The intensity of DyLight 549 was captured before and after DyLight 649 was photobleached. The measured FRET efficiency (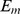) was calculated using the intensity of DyLight 549 before (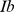) and after (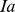) DyLight 649 photobleaching (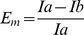 ). The averaged values of two measurements were plotted with standard deviations. (B) After normalization, the measured FRET efficiencies match those calculated based on the Flory radius (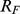) of the PEG polymers. The 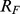 of the PEG polymer of 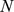
subunits and unit length 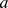 was calculated using 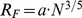, where 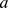 is 0.28 nm [32]. Theoretical FRET efficiency (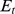) was calculated using the equation 
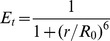
, where the Förster distance (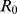) of the DyLight 549-DyLight 649 donor-acceptor pair is 5 nm and the distance between the pMHC ligand and streptavidin 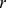 is 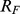 of the PEG polymer plus the pMHC radius of 2 nm. The FRET efficiencies were normalized by dividing the FRET efficiencies by the FRET efficiency of PEG 88.

IEkMCC-PEG conjugates, each with a biotin at the free end of the PEG polymer, were tethered on plastic surfaces covalently coated with streptavidin (Fig. 1). To analyze the average distance of IEkMCC protein to the surface, the efficiency of fluorescence resonance energy transfer (FRET) between DyLight 549-labeled IEkMCC and DyLight 649-labeled streptavidin was measured (Fig. S2). As shown in Fig. 3A, high FRET efficiency was observed between IEkMCC and streptavidin linked with the shortest linker PEG 88. FRET efficiency gradually decreased with increasing length of the PEG linker. FRET efficiency was no longer measurable when linkers longer than PEG 7500 were used.

The acceptor photobleaching-based FRET assay used here compares the fluorescence intensity of FRET donor (DyLight 549) before and after the photobleaching of the FRET acceptor (DyLight 649). The photobleaching step takes many minutes. Therefore, the FRET efficiency indicates an average distance between IEkMCC and streptavidin, rather than an instantaneous distance. The FRET results are consistent with the average shape of the PEG polymer as a sphere of Flory radius in an aqueous medium. Indeed, after normalization against the FRET efficiency of PEG 88, the measured FRET efficiencies of PEG polymers matched closely with the ones calculated based on the Flory radii of the polymers (Fig. 3B). It should be noted, however, that IEkMCC proteins are not fixed at a particular distance from the surface for each surface-tethered pMHC. Behaving similarly to an ideal chain [14], a PEG polymer is highly dynamic in solution. The IEkMCC protein can therefore be positioned at any distance within the contour length (fully extended length) of the polymer at any given moment. Taken together, the FRET data indicate that IEkMCC ligands tethered on the surface via PEG polymers behaved as predicted based on known PEG behavior.

### T Cell Activation by pMHC Ligands Tethered to a Surface

**Figure 4:**
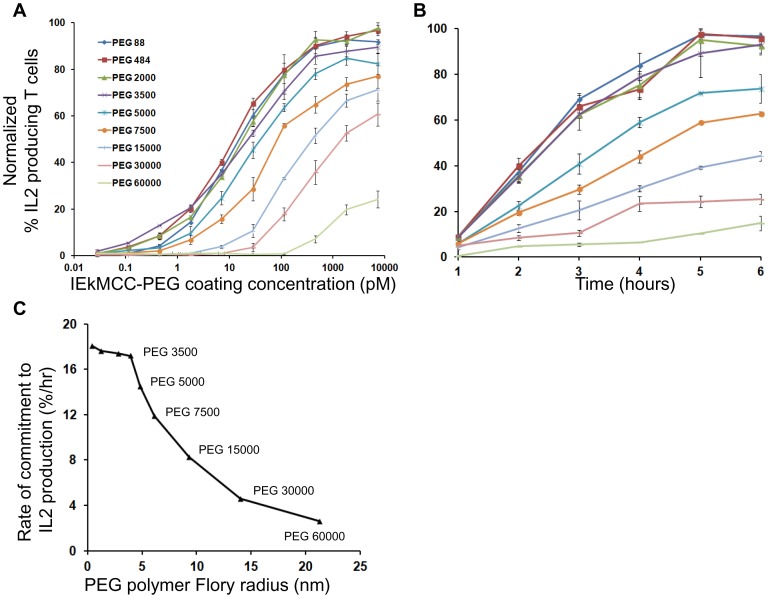
T cell activation by IEkMCC tethered with PEG polymers of different lengths. (A) T cell IL2 production in response to IEkMCC-PEG ligands of varying coating densities after 6 hours of stimulation. Data are representative of three independent experiments. The percent of T cells producing IL2 was determined by intracellular staining and flow cytometry. Three experiments using T cells from three different mice were performed (see Fig. S8 for flow cytometry plots). The percent of T cells producing IL2 was normalized to the highest value in each experiment. The data points are averages of the normalized values with standard errors of the means. (B) The rate of T cell response to IEkMCC ligands tethered with PEG polymers of different lengths. T cell IL2 production in response to stimulation on 96 well plates coated with 110 pM IEkMC-PEG ligands. T cells were harvested every hour for 6 hours and levels of IL2 expression were assayed by flow cytometry. Three experiments using T cells from three different mice were performed (see Fig. S9 for flow cytometry plots). The percent of T cells producing IL2 was normalized to the highest value in each experiment. The data points are averages of the normalized values with standard errors of the means. (C) The rates of T cell IL2 responses to IEkMCC ligands tethered with PEG polymers were extracted from the slope of linear fitting curves in Fig. 4B and plotted against the Flory radius of the polymers. The linear regressions and equations for deriving the rates are shown in Fig. S7.

To determine how T cell activation is affected by ligand-surface tethering by PEG polymers, IEkMCC ligands tethered to a surface with nine different PEG polymers were used to stimulate IEkMCC-specific 5C.C7 T cells. The surface densities of ligands tethered with different polymers were comparable when detected using the IEk-specific antibody 14-4-4s (Fig. S3). T cells were stimulated in ligand-coated wells for 6 hours, and IL2 production was measured by intracellular staining and flow cytometry. Dose-dependent responses were observed for all linkers, as well as an inverse correlation between the percentage of IL2 producing cells and linker length above a critical size (Fig. 4A). IEkMCC tethered with PEG 88, PEG 484, PEG 2000, and PEG 3500appeared to stimulate IL2 production with similar and high efficiency. Linkers larger than PEG 3500 showed a gradually decreasing ability to stimulate IL2 production, although IEkMCC tethered with the longest linker, PEG 60000 (contour length = 380 nm), was still capable of inducing low levels of IL2 production at high coating concentrations (>100 pM).

IEkMCC with the shortest linker, PEG 88, had the same efficiency as IEkMCC without a PEG linker but biotinylated at the lysine residue of a c-terminal AviTag sequence (IEkMCC-bio) [15] (Fig. S4A). This was expected, since PEG 88 has a contour length of only 0.6 nm, or the length of about 5 peptide bonds (0.13 nm). Consistent with the dispensable role of costimulatory molecules in activating pre-activated T cells [16], [17], addition of costimulatory molecule B7.1 to the surface did not enhance the ability of IEkMCC-PEG 88 to induce cytokine production (Fig. S4A). Addition of free polymers did not affect T cell activation by pMHC-bio (Fig. S4B). The maleimide group at one end of the free polymer was hydrolyzed to abolish its reactivity. The biotin group on the other end allows binding to streptavidin on the plate. T cells adhered well to plate surfaces coated with all IEkMCC-PEGs regardless of PEG length (Fig. S5). T cell adhesion may have been facilitated by binding to RYD sequences in streptavidin, which mimic the integrin-binding RGD sequence [18].

T cell responses to different concentrations of ligands at a given time point may not accurately reflect the relationship between T cell response and PEG linker length. It is possible, for example, that similar responses to the shorter polymers are due to saturation of the response at the time of stimulation termination. In other words, these polymers may have stimulated T cells to similar levels of response at a particular end point, but with a different rate. To explore this, T cells were stimulated with a fixed concentration of pMHC ligand and IL2 production assayed at different time points. As shown in Fig. 4B and 4C, the rate of T cells committing to IL2 production depended on the PEG linker length in a manner similar to that observed for dose response. Taken together, these results demonstrate that T cell responses inversely correlate with PEG polymer length, with the exception of PEGs smaller than PEG 5000.

### Kinetics of TCR Interaction with pMHC Tethered on a Surface by Molecular Dynamics Simulation

To explore how PEG length influences the kinetics of pMHC-TCR binding, we carried out coarse-grained Molecular Dynamics (CG-MD) simulations to examine the binding *in silico*. As shown in Fig. S6, the interaction between PEG-tethered pMHC ligand and TCR on a cell surface was represented by the interaction between the free end of a fully flexible PEG polymer (with another end fixed in position) and a patch of DMPC lipid bilayer [19], [20]. A weak binding potential mimicking the affinity of pMHC-TCR binding (∼10 µM) was added between the free end of the PEG polymer and the lipid bilayer. Binding was defined as an event in which the mobile tail bead of the polymer moves within a 1 nm cutoff distance from any portion of the lipid bilayer. The PEG-bilayer system was simulated as a function of PEG length to examine how polymer length (chain entropy) affects rate of binding. PEG 4000, PEG 10000 and PEG 20000 were chosen mainly because of the clear PEG length effect on T cell activation in this MW range. Also, MD simulations of longer PEG lengths are unrealistic due to their long relaxation times (≥200 ns). Consideration of protein geometry was omitted to make the simulation feasible with available computational resources. While this simplification hinders derivation of absolute values for binding kinetics, the relative impact of polymer length on binding kinetics may be assessed. Details of the multiple-replica CG-MD [21], [22] simulations accelerated by graphics processing units (GPU’s) [23] are fully described in Text S1.

**Figure 5:**
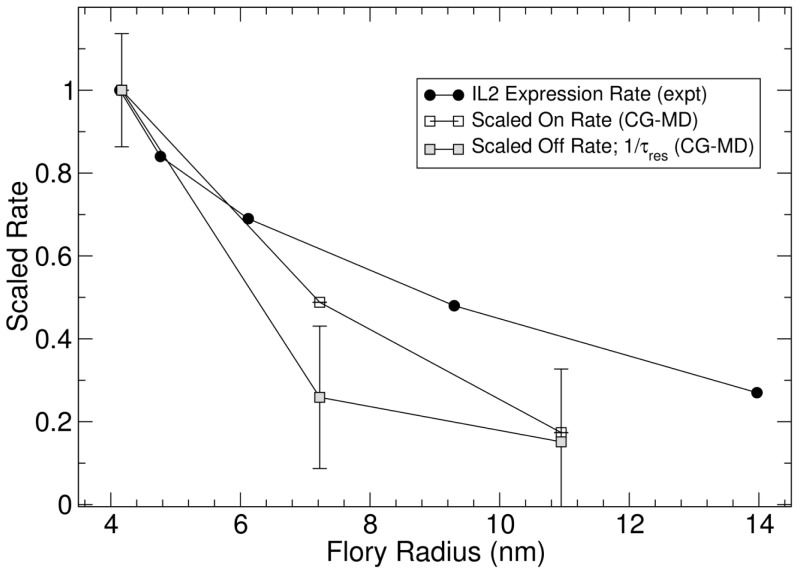
The impact of polymer length on pMHC-TCR binding kinetics based on CG-MD simulation. The on-rates and off-rates derived from the simulation for the PEG 4000, PEG 10000 and PEG 20000 were scaled against the data for the PEG 4000 and plotted as a function of the polymer Flory radius. To display the relationship between binding rates and TCR triggering efficiency, the experimentally determined IL2 expression rates for PEG 3500, PEG 5000, PEG 7500, PEG 150000 and PEG 30000 shown in Fig. 4A were scaled against PEG 3500 and plotted as a function of the polymer Flory radius.

To assess the relative impact of polymer length on binding kinetics, we scaled the binding rates of PEG 10000 and PEG 20000 against the rates of PEG 4000. As shown in Figure 5, both the on-rate and off-rate of the binding decreased with linker length. The decrease of the on-rate is possibly due to steric effects of long polymers limiting access to binding sites, as shown in Text S1. The decrease of the off-rate with polymer length may be explained by the lower entropic force applied to the binding by longer polymers, since the entropic force of the polymer is inversely proportional to the length of the polymer chain. The decrease of overall binding rate with polymer length can also be justified in a conceptual context by considering the longer relaxation time of the longer polymers (Text S1). We also estimated the effective force applied to the pMHC-TCR binding from the CG-MD simulations using the potential of mean force acting at the most probable binding distance. Around 40 pN of force was applied by PEG 4000 and the force decreased slightly for longer polymers. It has been shown that 40 pN is large enough to initiate protein conformational changes [24], [25]. Together, our CG-MD results suggest that the ability of tethered pMHC to activate T cells correlates with the rates of TCR binding. In addition, pMHC-TCR may sustain a significant amount of mechanical force derived from polymer entropic energy.

## Discussion

To investigate how 2D pMHC-TCR interaction contributes to TCR triggering, we gradually increased the range of motion of pMHC ligands in the third dimension using flexible PEG polymers. Our approach differs from previous studies where pMHC or scFv of anti-CD3 antibody were fused on top of CD4 and other transmembrane proteins [26], [27]. The TCR binding sites of these molecules are elevated from the cell surface at a fixed distance by the added protein domains. Their movement in the third dimension, however, still fully depends on the movement of the cell membrane. Therefore, these molecules still interact with TCR in a strictly 2D fashion. In contrast, pMHC ligands in our system are tethered to a surface through flexible PEG linkers. Although these polymers take on a spherical equilibrium configuration in an aqueous environment, their chain units move much more freely than the amino acid residues in a folded protein molecule. For instance, PEG 7500 and pMHC were eluted at similar volumes from gel filtration columns, indicating their similar hydrodynamic radii. When attached to a surface on one end, however, the free end of PEG 7500 polymer could be at any position within the contour length of the polymer (∼48 nm) at a given moment, whereas the distal end of pMHC would stay 5 nm above the surface. On the other hand, it should be noted that the “free” end of the polymer does not diffuse freely like an untethered molecule. Its positional dynamics is determined by thermal motion, which will be affected by the chain entropy of the polymer.

The interaction between TCR and pMHC tethered to a surface with PEG polymers therefore must have characteristics of both 2D and 3D interactions. Ideally, their binding kinetics should be determined experimentally. This, however, is technically highly challenging and is beyond the scope of this paper. Unlike 3D interaction kinetics that can be routinely characterized with the mature surface plasmon resonance technology using commercially available systems, such as Biacore, 2D kinetics characterization is still a developing field of research. Different methodologies often give dramatically different results for similar ligand-receptor interactions. For example, 2D pMHC-TCR interactions have been characterized using laminar flow chamber [9], fluorescence microscopy imaging [10], and micropipette and biomembrane force probes [11]. The 2D off-rates obtained varied from being similar to the 3D off-rate [9], to about ten-fold higher than the 3D off-rate [10], to a thousand fold higher than the 3D off-rate [11]. To the best of our knowledge, no attempt has been made to date to experimentally measure protein binding behavior in a system that tethers ligands on a 2D surface using flexible polymer linkers to better understand the parameters governing 2D interactions in T cell activation.

CG-MD simulation offered an alternative approach for assessing the influence of PEG polymers on pMHC-TCR binding at the T cell-surface interface. CG-MD simulations have proven useful for understanding complex molecular level phenomena including pMHC-TCR interactions [28], [29], [30]. The combination of state-of-the-art GPU computing and a CG-MD approach in our study enabled us to simulate nearly the entire range of PEG lengths to characterize the kinetics of pMHC-TCR binding. Our simulation setup is based on a robust model for the interaction between a free end of PEG polymer and the lipid bilayer, with a binding potential added to match the affinity of pMHC-TCR interaction. It should be noted, however, that certain assumptions and simplifications were required to make the approach computationally feasible. First, the physical dimension and geometry of the pMHC and TCR proteins were not considered. Second, a binding event is arbitrarily defined as when the binding partners are within 1 nm of distance. And third, the distance between the two surfaces were fixed. With these simplifications, it is difficult to reliably derive realistic absolute values for kinetics parameters. The qualitative influence of PEG length on binding rates, however, may be reasonably assessed. The simulation showed that the on-rate and off-rate of pMHC-TCR binding decreases with the length of the linker. Also, the PEG polymer entropy applies a significant amount of force to the pMHC-TCR binding to better understand the parameters governing 2D interactions in T cell activation.

Our T cell activation experiments show that T cell responses to PEG-tethered ligands in terms of IL2 production displayed two distinct phases (Fig. 4C). pMHCs tethered by the four smallest PEGs, PEG 88, 484, 2000, and 3500, stimulated T cells similarly and with high efficiency. At this small MW range, the effect of PEG polymer dynamics is relatively small, and the kinetics of pMHC-TCR interaction is dominated by factors associated with intrinsic cell membrane dynamics. The relatively fixed “upright” orientation of pMHC ligands tethered with these short linkers may also play a dominant role in the kinetics of pMHC-TCR binding. In addition, we cannot exclude the possibility of TCR crosslinking for these short polymers, especially at high ligand density. Starting with PEG 5000, T cell responses gradually deceased with increasing PEG linker length. T cells responded to the largest PEG 60000 polymer, although only at high coating concentrations. In addition to IL2 production, we attempted to assay tyrosine phosphorylation using anti-phosphotyrosine antibodies and flow cytometry. The differences between ligands with different polymer lengths, however, could not be clearly resolved due to weak staining.

The results of our study are not consistent with the receptor crosslinking model or the kinetic segregation model. pMHCs tethered with PEG 2000 (contour length = 12.7 nm) or longer were able to trigger TCR. It is unlikely that TCR was triggered through TCR crosslinking by dimeric pMHCs. It has been shown that for activation through pMHC dimerized with linkers, the contour length of the linkers must be less than 10 nm [31]. Moreover, T cells were activated by low densities of pMHCs on a surface that would have only a small probability of forming dimers (Text S2). Our results do not support or disprove the co-receptor crosslinking model. Longer polymers might reduce pMHC potency by interfering with binding between pMHC and co-receptor. We think it is more likely, however, that the polymers interfere with the pMHC-TCR-CD4 three-party binding as a whole, given the closeness of the binding sites. The kinetic segregation model of TCR triggering relies on pMHC-TCR interaction at a tight space between two closely aligned membranes for the exclusion of large tyrosine phosphatase molecules such as CD45. In our study, pMHC ligands tethered through flexible PEG polymers such as PEG 5000 and longer should be able to interact with TCRs on relatively distant T cell plasma membranes. The gap between two membranes in this case should be able to accommodate CD45 and not cause its segregation with TCRs. Moreover, our CG-MD simulation results suggest that ligands with longer polymers bind TCRs more stably but trigger TCR less efficiently. This contradicts the kinetic segregation model, where TCR triggering depends on stable pMHC-TCR interaction [3].

Our results are consistent with the receptor deformation model of TCR triggering. In this model, pMHC-TCR bindings are pulled apart, or ruptured, by mechanical forces associated with the dynamic T cell-APC interaction. Before each rupture, signaling is triggered via the TCR through receptor deformation [5]. The overall triggering efficiency is determined by the frequency of rupture events, which is in turn determined by the on-rate and off-rate of binding. Higher rupture frequency facilitated by faster on-rate and off-rate translates to better integration of signals from multiple rupture events, thus, higher TCR triggering efficiency. While our work does not provide direct evidence for TCR triggering by receptor deformation, our simulation suggests that TCRs are subjected to mechanical forces derived from PEG polymer entropy. In addition, the experimental and simulation data show that pMHC ligands tethered with shorter polymers have higher on-rates and off-rates and trigger TCR more efficiently (Fig. 5). The correlation between binding rates and TCR triggering efficiency in our study is supported by a recent study that experimentally measured the 2D on-rates and off-rates of different pMHC-TCR binding pairs using micropipette and biomembrane force probes [11]. Furthermore, this study showed that 2D off-rates of pMHC-TCR binding were 30 to 8300-fold faster than the 3D off-rate [11], strongly suggesting that pMHC-TCR binding is under mechanical stress at the T cell-APC interface.

In summary, using pMHC ligands tethered to a surface with flexible PEG polymers, our study sheds light on the mechanism of the TCR triggering from a unique perspective. This approach of altering the 2D pMHC-TCR interaction should continue to offer valuable insights, in conjunction with methods to experimentally determine the kinetics of TCR binding to surface-tethered pMHC ligands.

## Materials and Methods

### Mice, cells and reagents

B10.BR H-2k mice and 5C.C7 TCR transgenic mice were purchased from Jackson Laboratories and Taconic, respectively. To generate T cell blasts, splenocytes from 5C.C7 TCR transgenic mice and irradiated splenocytes from B10.BR mice were mixed in complete Click’s medium (Irvine Scientific, Santa Ana, CA) supplemented with 50 U/ml IL2 and 50 µM moth cytochrome c peptide. T cell blasts were used on day 7 to day 10 post stimulation. All animal experiments were approved by the Institutional Animal Care and Use Committee (IACUC) of Nemours/A.I. duPont Hospital for Children. All efforts were made to minimize the number of animals used and their suffering. Heterobiofunctional PEG 88 and PEG 484 linkers with a maleimide group at one end and a biotin group at the other end were from Pierce (Rockford, IL). All other heterobifunctional linkers with longer PEG linkers were purchased from Jenkem Technology USA (Allen, TX). EDC (1-Ethyl-3-[3-dimethylaminopropyl] carbodiimide Hydrochloride), Sulfo-NHS, TCEP (Tris(2-Carboxyethyl) phosphine Hydrochloride), streptavidin-DyLight 649, DyLight 549, and PBS (0.1 M phosphate and 0.15 M NaCl) were from Pierce (Rockford, IL). Brefeldin A, streptavidin, BSA (bovine serum albumin), MES (2-(*N*-morpholino)ethanesulfonic acid), and APTES (3-Aminopropyltriethoxysilane) were from Sigma-Aldrich (St. Louis, MO). DPBS (Dulbecco’s PBS) was from Invitrogen (Carlsbad, CA). 14-4-4s mAb was generated from a hybridoma kindly provided by John Kappler (National Jewish Health).

### Protein Preparation

Plasmid construct for expressing the extracellular domain of IEk with covalently linked MCC peptide was a gift from John Kappler (National Jewish Health). For conjugation with polymers, the construct was modified by PCR to add a cysteine at the C-terminus of the β chain. Baculoviruses were generated with the construct and BaculoGold Linearized baculovirus DNA (BD Biosciences, San Diego, CA) in Sf9 insect cells (Invitrogen, Calsbad, CA). High titer stocks of cloned recombinant baculovirus were used to infect Hi5 insect cells (Invitrogen, Calsbad, CA) cultured in spinner flasks. IEkMCC protein was purified from the supernatant of infected Hi5 cell cultures using an affinity chromatography column conjugated with 14-4-4s antibody. The protein was further purified using a Superdex 200 10/300 GL gel filtration column (GE Healthcare, Piscataway, NJ) before conjugating with PEG linkers. The design and production of IEkMCC with a C-terminal AviTag was described previously [15]. The protein was biotinylated at the lysine residue of the AviTag using BirA enzyme as described previously [15].

### Conjugate Formation and Purification

IEkMCC-Cys protein was first treated with 0.1 mM TCEP for 1 hour at room temperature to reduce the sulfhydryl group of the c-terminal free cysteine. The protein was then reacted with PEG polymer at 1∶20 molar ratio in PBS buffer pH 7.4 for 4 hours. For reactions with PEG 88, PEG 484 and PEG 2000, unreacted PEG linkers were separated from the conjugates and unreacted IEkMCC by gel filtration using a Superdex 200 10/300 GL gel filtration column, and the mixture of conjugates and unreacted IEkMCC protein was collected. The molar ratio of biotin to IEkMCC of the mixture was determined using a HABA (4'-hydroxyazobenzene-2-carboxylic acid)-based assay (Pierce, Rockford, IL). For reactions with PEG 3500, PEG 5000, and PEG 7500, IEkMCC-PEG conjugates were separated from unreacted IEkMCC and PEG linkers in one step using a Superdex 200 10/300 GL column. For reactions with PEG 15000, PEG 30000, and PEG 60000, unreacted PEG linkers were first eliminated using an affinity column conjugated with IEk specific antibody 14-4-4s. IEkMCC-PEG conjugates were then separated from unreacted IEkMCC by gel filtration using Superdex 200 10/300 GL.

### Covalent Coating of Streptavidin to Polystyrene Surfaces

96-well strip well tissue culture treated polystyrene plates (Corning, Lowell, MA) were treated with 5 mM EDC and 5 mM Sulfo-NHS in MES buffer (0.1 M MES, 0.15 M NaCl, pH 6.0) for 40 minutes to add NHS ester to the carboxyl groups on the polystyrene surface. After washing, 100 µg/ml streptavidin in PBS pH 7.4 was added and incubated overnight at room temperature. The plate was then blocked with DPBS with 1% BSA. The amount of bound streptavidin was determined as 40 ng per well using an assay based on the quenching of biotin-FITC (Invitrogen, Calsbad, CA) fluorescence by streptavidin binding.

### FRET Imaging and Analyses

For FRET imaging, DyLight 649-labeled streptavidin was coated on biotinylated plates (Pierce, Rockford, IL). IEkMCC-PEG(n)-Bio conjugates were labeled with DyLight 549 and coated on the plates via biotin-streptavidin binding. The bottom of the wells was imaged under a 60× water immersion objective on a Zeiss Axioplan 2 upright microscope. Using a 300 W xenon light source, we were able to achieve 95% photobleaching of DyLight 649 in about 5 minutes, and no photoconversion from DyLight 649 to DyLight 549 was observed (Fig. S2C). The intensity of DyLight 549 was recorded before and after DyLight 649 was photobleached. All images were analyzed with SlideBook software (Intelligent Imaging Innovations, Denver, CO).

### T Cell Stimulation and Flow Cytometry

IEkMCC-PEG conjugates in DPBS with 1 mg/ml BSA were incubated in streptavidin-coated wells overnight at 4°C. Plates were washed, and 2.5×10^5^ T cells in complete Click’s medium were added. For IL2 production, 20 µg/ml brefeldin A was added to the medium. After stimulation, T cells were harvested, fixed with 3% formaldehyde in DPBS, permeabilized with DPBS buffer containing 1% BSA and 0.1% saponin, and stained with APC-labeled anti-mouse IL2 antibody JES6-5H4 (Biolegend, San Diego, CA).

### Coarse-grained Molecular Dynamics Simulation

MD simulations were performed on the sticky membrane PEG systems, in which polymer length was varied from 4000 to 20000 Da. Multiple replica random-walker MD simulations were employed and run in the NPT ensemble using a GPU-accelerated CG model at a temperature of 300 K and pressure of 1 atm. Individual simulations were run for 500 ns each, and a total of 20 replicas were used per system, producing 10 microseconds of aggregate trajectory data per polymer. Details of these simulations and the resulting theoretical calculations can be found in Text S1.

## Supporting Information

Figure S1 Characterization of IEkMCC proteins. (A) Purified IEkMCC protein with a free c-terminal cysteine was analyzed by SDS-PAGE and Coomassie Blue staining. The denatured protein migrated as two distinct bands with molecular weights consistent with the α and β chains. (B) Purified protein was analyzed by gel filtration chromatography using a Superdex 200 10/300 GL column. The protein was eluted as a single peak with a molecular weight of ∼55 KDa.
(TIF)

Figure S2 Measurement of FRET between surface-tethered IEkMCC ligands and streptavidin on a plastic surface. (A) Schematic of FRET setup. The IEkMCC protein was labeled with DyLIght 549 (red star). The streptavidin on a plastic surface was labeled with DyLight 649. (B) Acceptor photobleaching FRET for linkers PEG 88 and PEG 7500. For the short PEG 88 linker, a significant increase in DyLight 549 intensity was seen after DyLight 649 was photobleached. For the long PEG 7500 linker, only a slight increase in DyLight 549 intensity was observed after DyLight 649 photobleaching. (C) Photobleaching of DyLight 649 does not lead to photoconversion to DyLight 549. Streptavidin labeled with DyLight 649 (Cy5-like dye) was continuously imaged at both DyLight 549 and DyLight 649 channels with 1 s exposure time and 0 second intervals.
(TIF)

Figure S3 Similar densities of IEk-MCC tethered on plastic through PEG linkers of different lengths. IEkMCC-PEG conjugates at the indicated concentrations were incubated overnight at 4°C on streptavidin-coated 96-well plates. After washing, IEkMCC was detected using the IEk-specific antibody 14-4-4s and goat anti-mouse HRP.
(TIF)

Figure S4 T cell activation by IEkMCC-PEG 88 is independent of PEG tether, constimulatory molecules, or free PEG linkers. Streptavidin plates were coated with IEkMCC-PEG 88 or IEkMCC-bio (IEkMCC without a PEG linker but biotinylated at the lysine residue of a c-terminal AviTag sequence) at indicated concentrations. To test the effect of costimulatory molecules, plates coated with IEkMCC-PEG 88 were washed and further coated with B7.1-Fc fusion protein (1 nM; R&D Systems, biotinylated at 2.3 biotins per molecule). T cell IL2 production was measured by intracellular staining and flow cytometry after 6 hrs of stimulation. (B) IEkMCC-bio without PEG linker (7.2 nM) was anchored on streptavidin coated plates by incubating overnight at 4°C. Polymers (200 pM) were then added for 1 hr at room temperature. The PEG polymers were pre-incubated in PBS overnight at room temperature to hydrolyze the maleimide group. T cells were added to the washed plates and incubated for 6 hrs. Percent of IL2 producing cells was determined using intracellular cytokine staining and flow cytometry. The data points are averages of the values from replicate samples with standard deviations.
(TIF)

Figure S5 T cell adhesion to plates with tethered ligands is independent of linker length. T cells were added to plates coated with IEkMCC-PEG88, IEkMCC-PEG 5000, or IEkMCC-PEG 60000 at 7.2 nM. Cells were imaged 1 hr later using Evos microscope (Life Technologies) with a 20× objective.
(TIF)

Figure S6 Snapshot of the molecular dynamics simulation. The PEG-bilayer system was simulated as a function of PEG length (from 4000 to 20000 Dalton as shown in red) with a distance of 9 nm between the fixed end of the polymer and the center of the bilayer to examine how polymer length (chain entropy) affects both the rate and affinity of binding.
(TIF)

Figure S7 Linear regression trend lines and equations are displayed for Fig. 4B to show the rate of T cell commitment to IL2 production.
(TIF)

Figure S8 Flow cytometry plots for the three experiments done for Fig. 4A. At least 5000 live cells were collected for each sample. Note that in Experiment #1, IEkMCC-PEG 88 was not assayed.
(TIF)

Figure S9 Flow cytometry plots for the three experiments done for Fig. 4B. At least 5000 live cells were collected for each sample. Note that only three time points were assayed in Experiment #1.
(TIF)

Text S1 CG-MD Simulation.
(DOC)

Text S2 Calculation of the number of pMHC-PEG-bio bound to each streptavidin on a plastic surface.
(DOC)
