# Supplementary figures and images for: TCR Triggering by pMHC Ligands Tethered on Surfaces via Poly(Ethylene Glycol) Depends on Polymer Length
Source: PLoS One. 2014 Nov 10;9(11):e112292. doi: 10.1371/journal.pone.0112292 (PMC4226474; doi:10.1371/journal.pone.0112292)

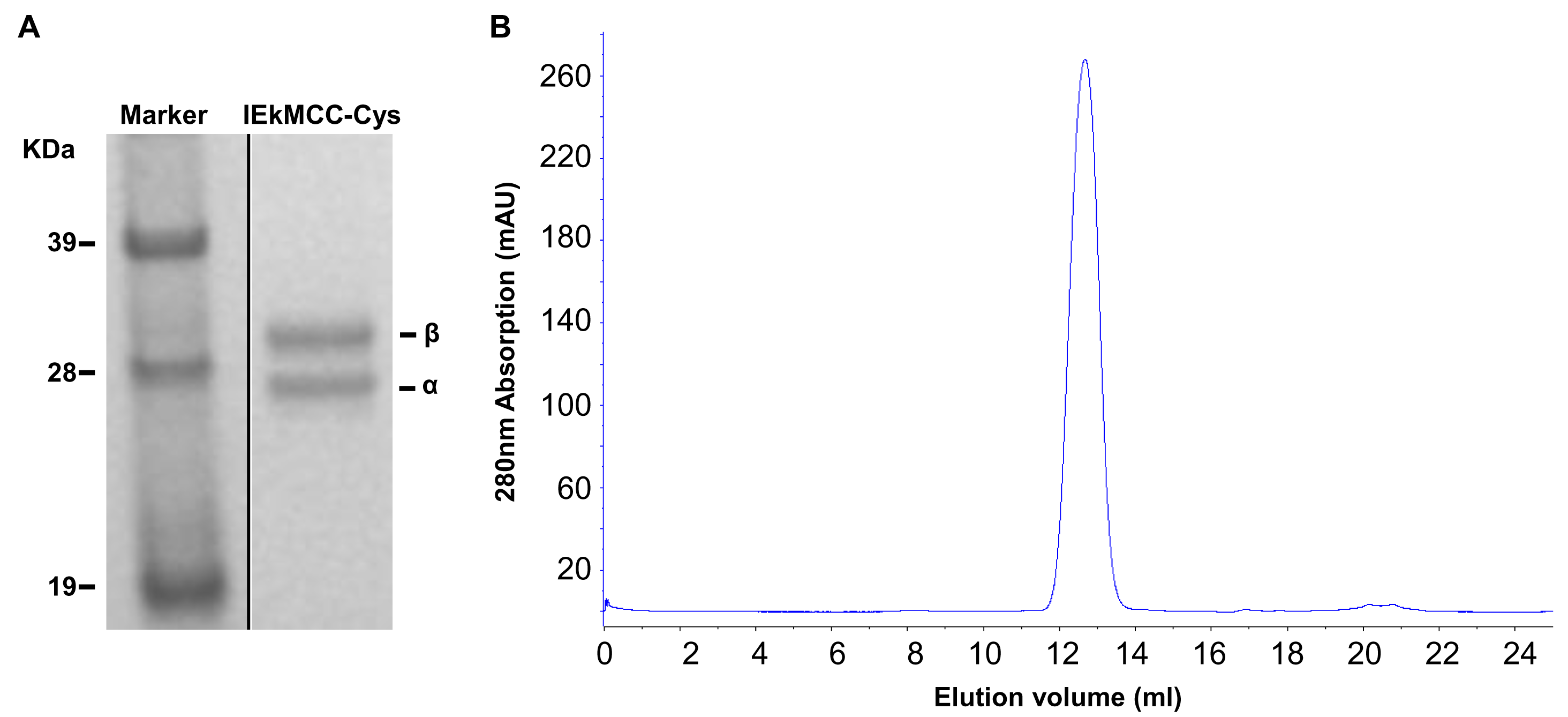

Supplement: Figure S1 — Characterization of IEkMCC proteins. (A) Purified IEkMCC protein with a free c-terminal cysteine was analyzed by SDS-PAGE and Coomassie Blue staining. The denatured protein migrated as two distinct bands with molecular weights consistent with the α and β chains. (B) Purified protein was analyzed by gel filtration chromatography using a Superdex 200 10/300 GL column. The protein was eluted as a single peak with a molecular weight of ∼55 KDa. (TIF) [file pone.0112292.s001.tif]

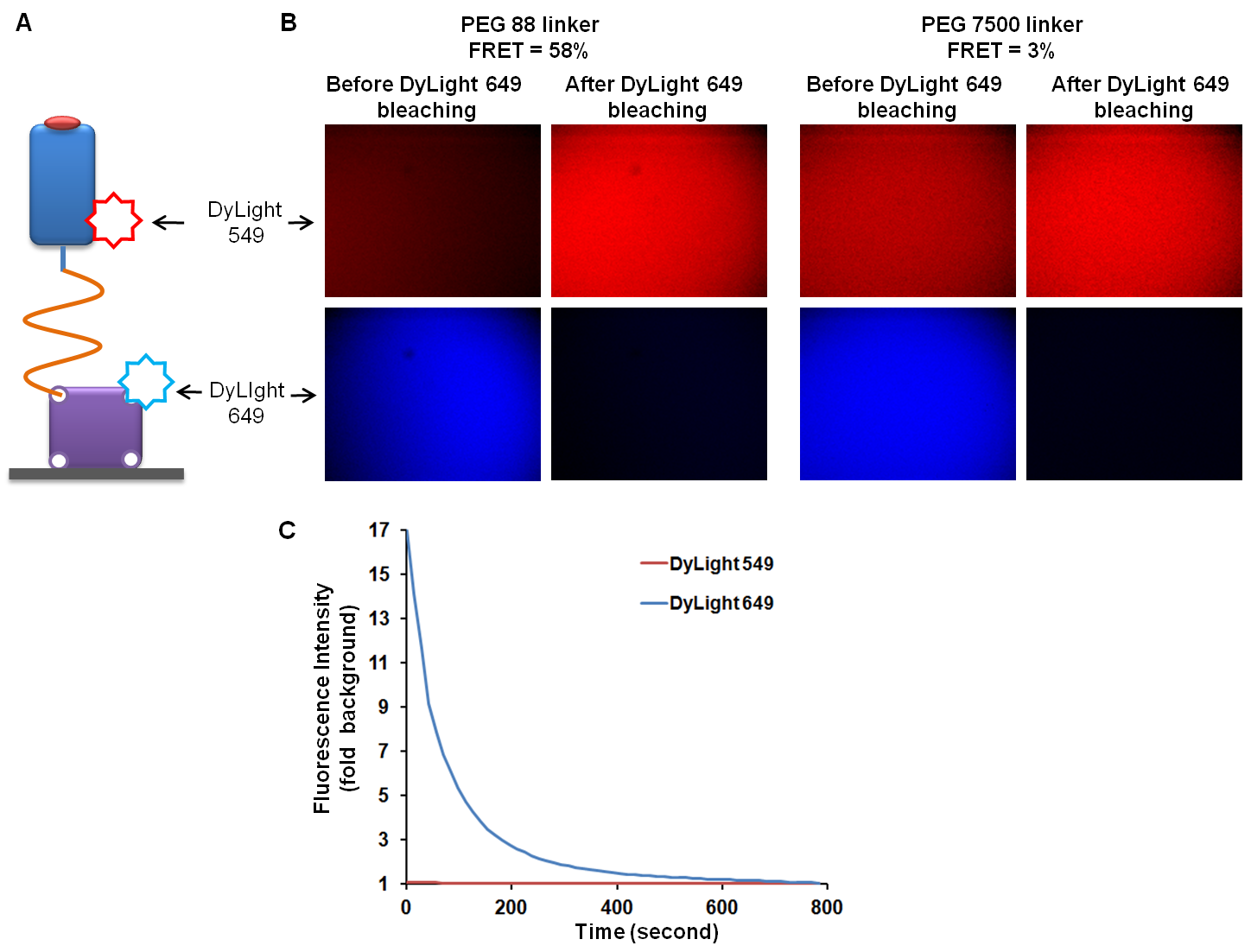

Supplement: Figure S2 — Measurement of FRET between surface-tethered IEkMCC ligands and streptavidin on a plastic surface. (A) Schematic of FRET setup. The IEkMCC protein was labeled with DyLIght 549 (red star). The streptavidin on a plastic surface was labeled with DyLight 649. (B) Acceptor photobleaching FRET for linkers PEG 88 and PEG 7500. For the short PEG 88 linker, a significant increase in DyLight 549 intensity was seen after DyLight 649 was photobleached. For the long PEG 7500 linker, only a slight increase in DyLight 549 intensity was observed after DyLight 649 photobleaching. (C) Photobleaching of DyLight 649 does not lead to photoconversion to DyLight 549. Streptavidin labeled with DyLight 649 (Cy5-like dye) was continuously imaged at both DyLight 549 and DyLight 649 channels with 1 s exposure time and 0 second intervals. (TIF) [file pone.0112292.s002.tif]

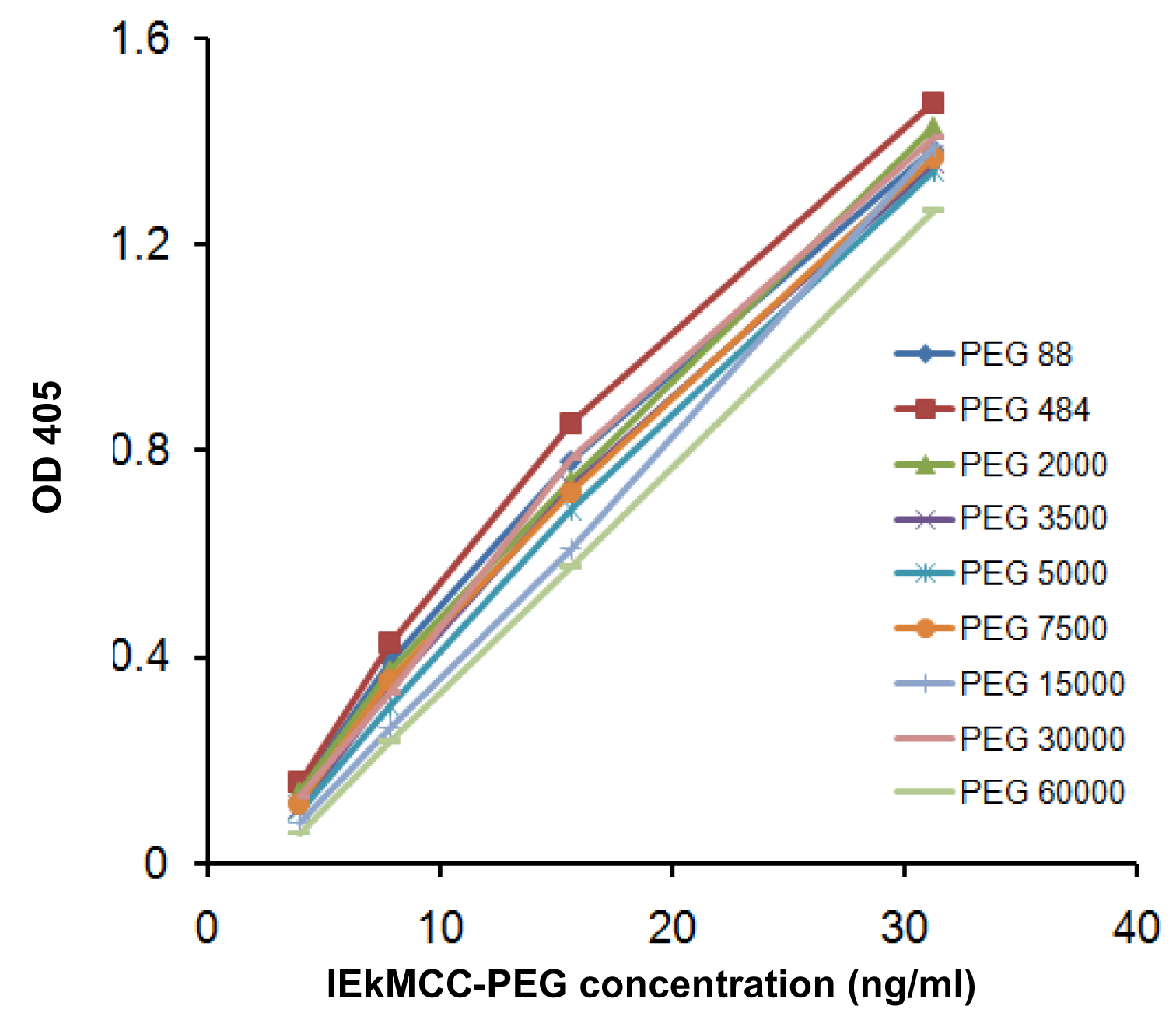

Supplement: Figure S3 — Similar densities of IEk-MCC tethered on plastic through PEG linkers of different lengths. IEkMCC-PEG conjugates at the indicated concentrations were incubated overnight at 4°C on streptavidin-coated 96-well plates. After washing, IEkMCC was detected using the IEk-specific antibody 14-4-4s and goat anti-mouse HRP. (TIF) [file pone.0112292.s003.tif]

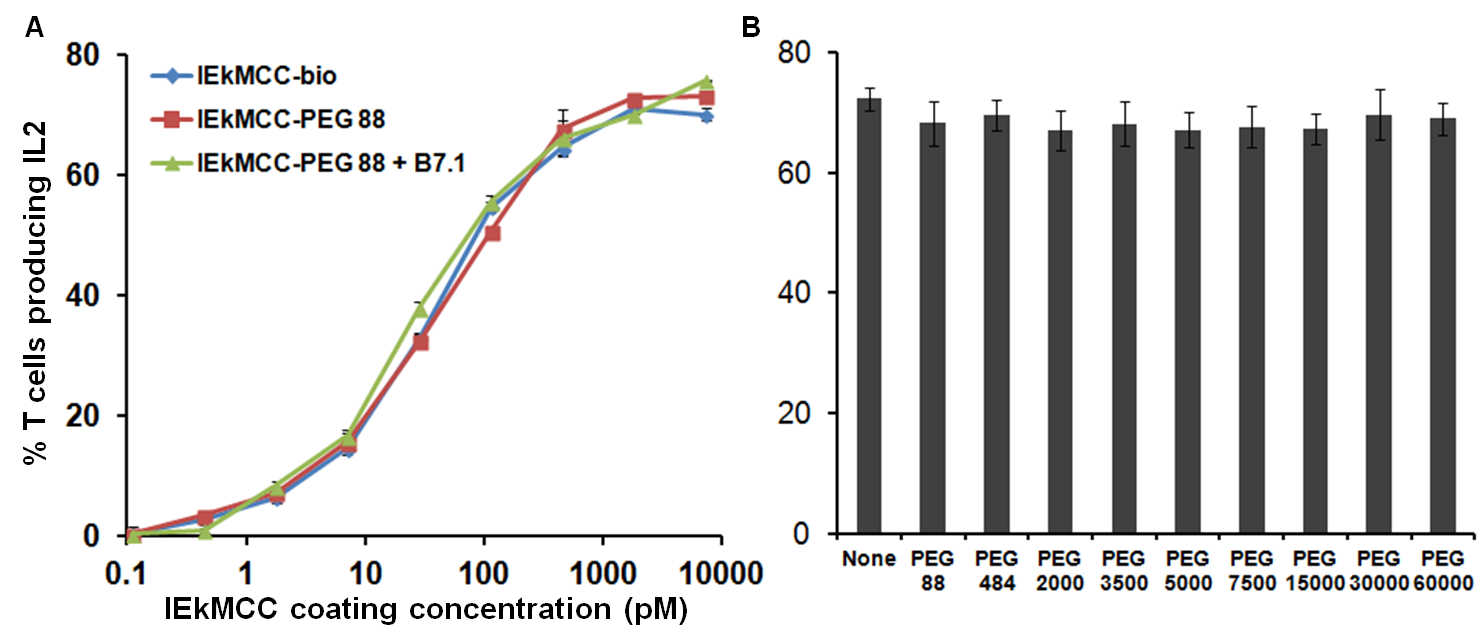

Supplement: Figure S4 — T cell activation by IEkMCC-PEG 88 is independent of PEG tether, constimulatory molecules, or free PEG linkers. Streptavidin plates were coated with IEkMCC-PEG 88 or IEkMCC-bio (IEkMCC without a PEG linker but biotinylated at the lysine residue of a c-terminal AviTag sequence) at indicated concentrations. To test the effect of costimulatory molecules, plates coated with IEkMCC-PEG 88 were washed and further coated with B7.1-Fc fusion protein (1 nM; R&D Systems, biotinylated at 2.3 biotins per molecule). T cell IL2 production was measured by intracellular staining and flow cytometry after 6 hrs of stimulation. (B) IEkMCC-bio without PEG linker (7.2 nM) was anchored on streptavidin coated plates by incubating overnight at 4°C. Polymers (200 pM) were then added for 1 hr at room temperature. The PEG polymers were pre-incubated in PBS overnight at room temperature to hydrolyze the maleimide group. T cells were added to the washed plates and incubated for 6 hrs. Percent of IL2 producing cells was determined using intracellular cytokine staining and flow cytometry. The data points are averages of the values from replicate samples with standard deviations. (TIF) [file pone.0112292.s004.tif]

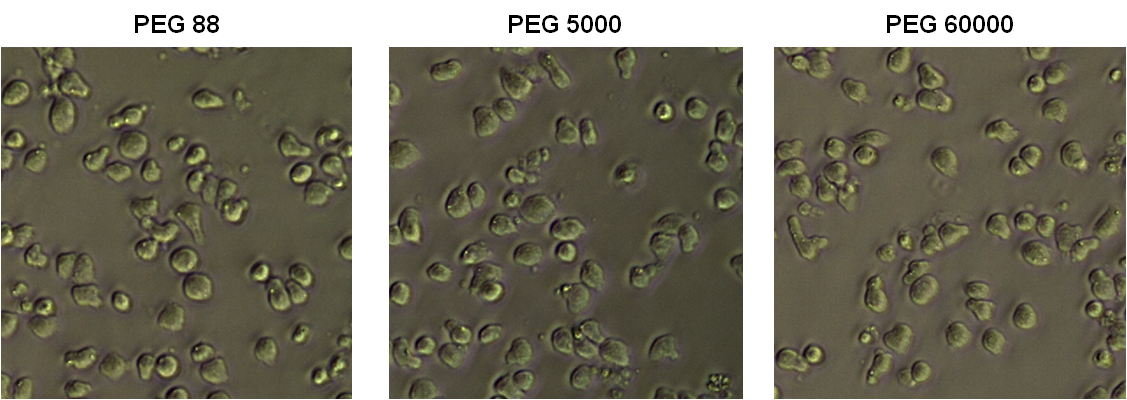

Supplement: Figure S5 — T cell adhesion to plates with tethered ligands is independent of linker length. T cells were added to plates coated with IEkMCC-PEG88, IEkMCC-PEG 5000, or IEkMCC-PEG 60000 at 7.2 nM. Cells were imaged 1 hr later using Evos microscope (Life Technologies) with a 20× objective. (TIF) [file pone.0112292.s005.tif]

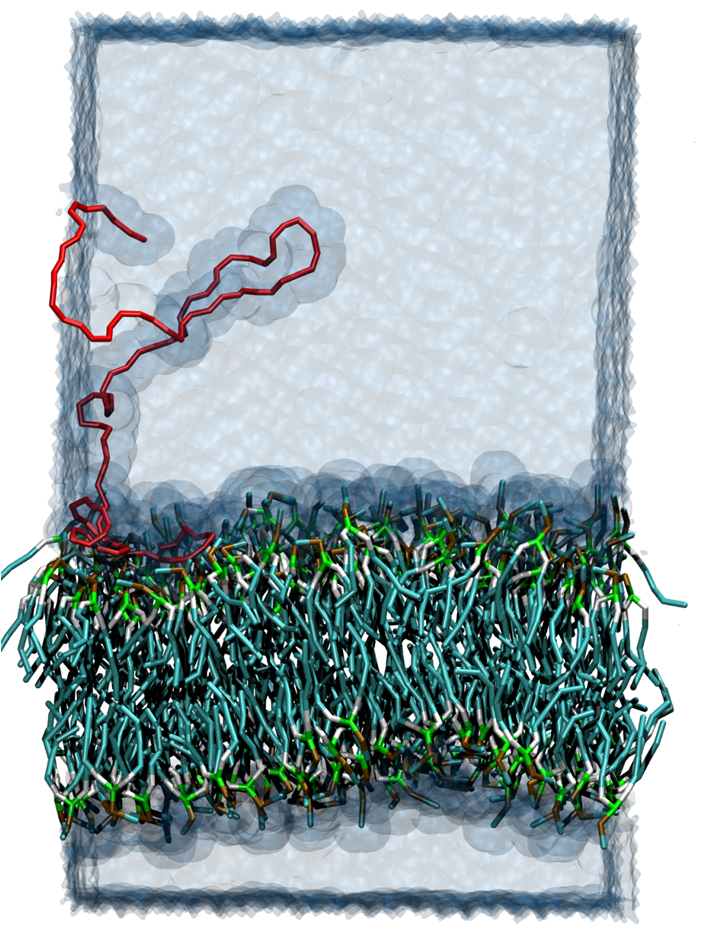

Supplement: Figure S6 — Snapshot of the molecular dynamics simulation. The PEG-bilayer system was simulated as a function of PEG length (from 4000 to 20000 Dalton as shown in red) with a distance of 9 nm between the fixed end of the polymer and the center of the bilayer to examine how polymer length (chain entropy) affects both the rate and affinity of binding. (TIF) [file pone.0112292.s006.tif]

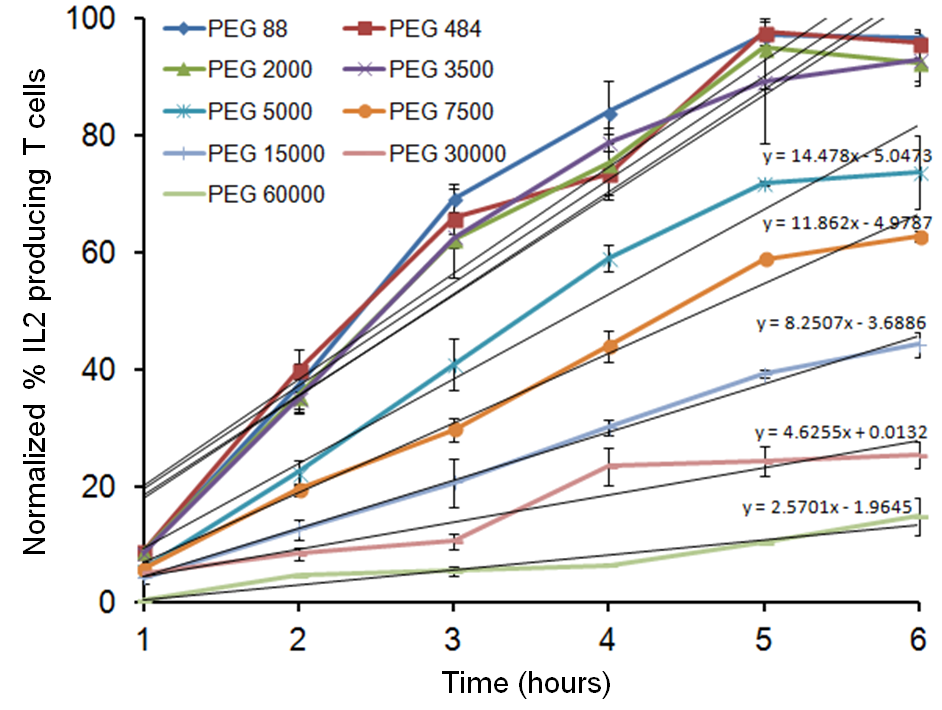

Supplement: Figure S7 — Linear regression trend lines and equations are displayed for Fig. 4B to show the rate of T cell commitment to IL2 production. (TIF) [file pone.0112292.s007.tif]

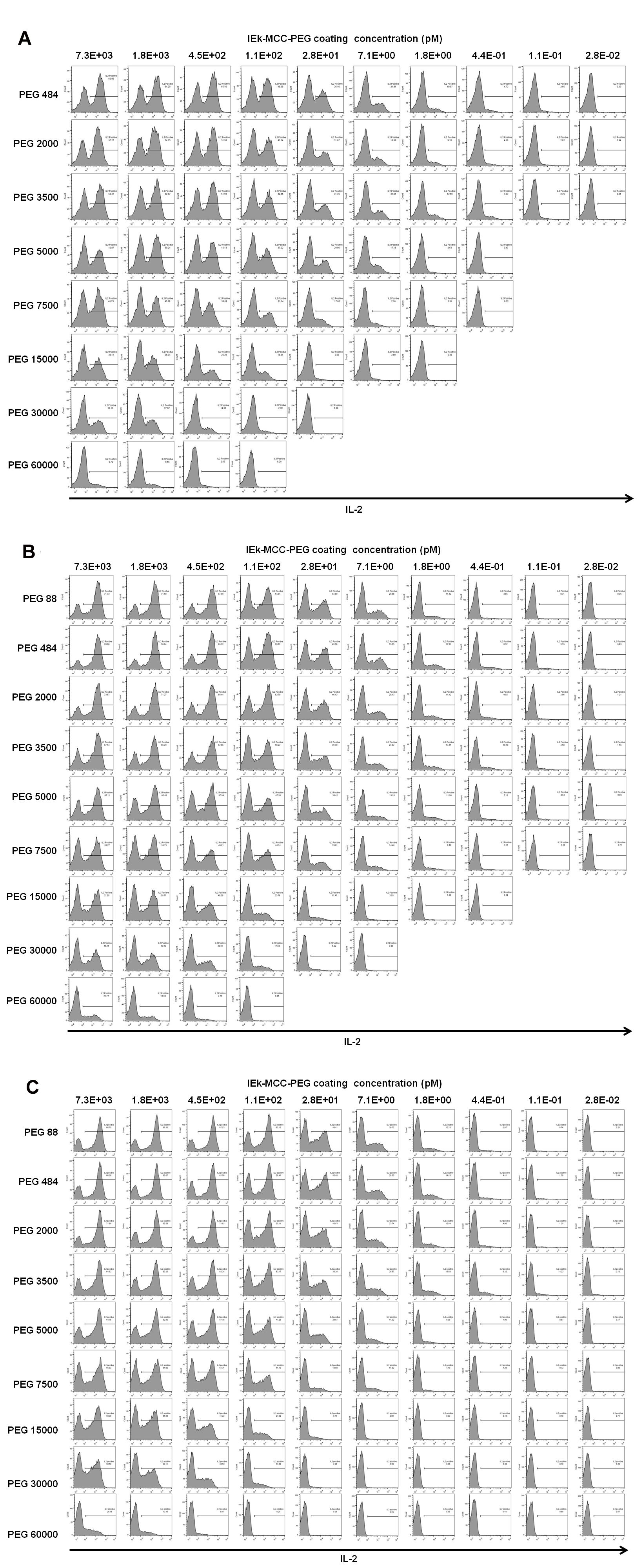

Supplement: Figure S8 — Flow cytometry plots for the three experiments done for Fig. 4A. At least 5000 live cells were collected for each sample. Note that in Experiment #1, IEkMCC-PEG 88 was not assayed. (TIF) [file pone.0112292.s008.tif]

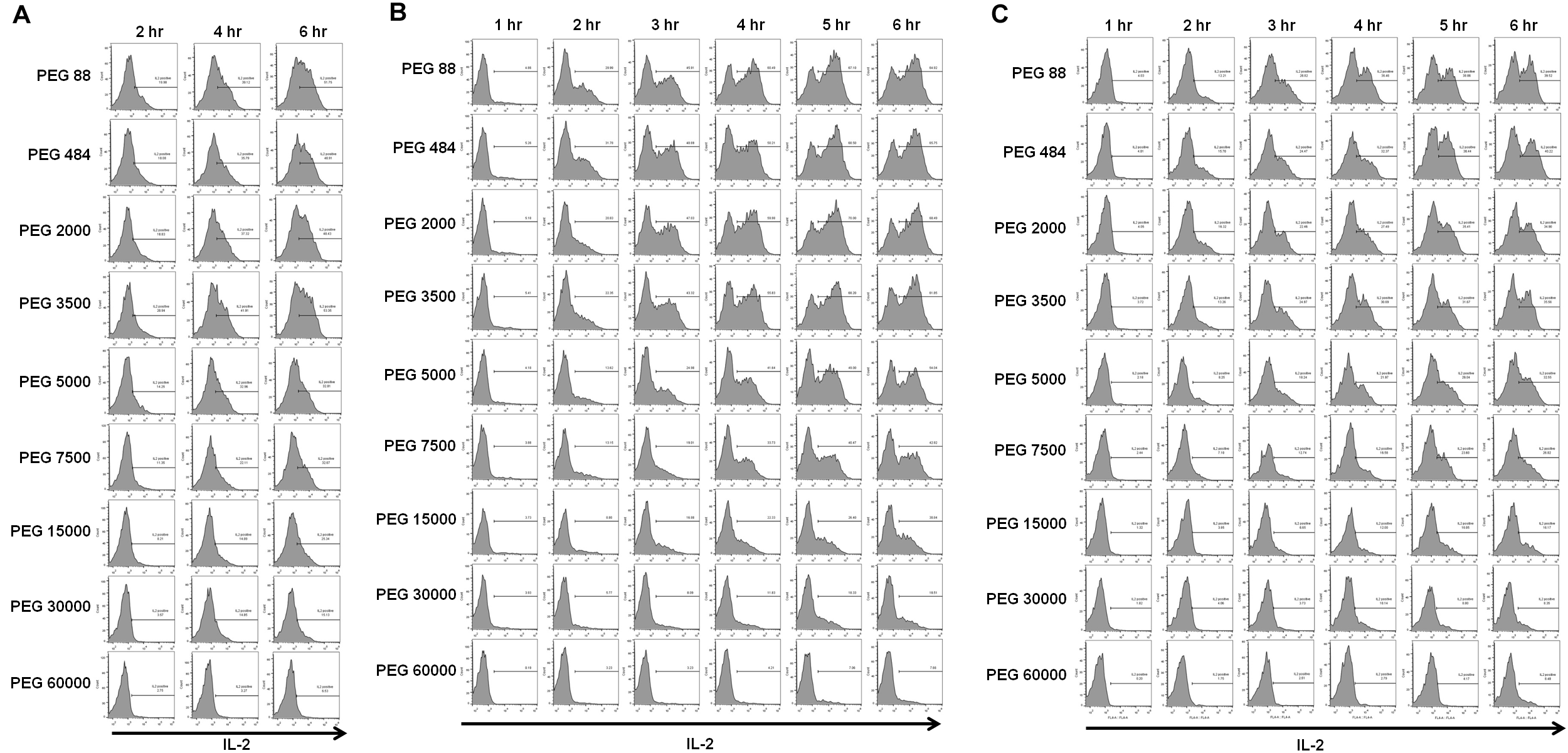

Supplement: Figure S9 — Flow cytometry plots for the three experiments done for Fig. 4B. At least 5000 live cells were collected for each sample. Note that only three time points were assayed in Experiment #1. (TIF) [file pone.0112292.s009.tif]
