# Supplementary material for: TCR Triggering by pMHC Ligands Tethered on Surfaces via Poly(Ethylene Glycol) Depends on Polymer Length
Source: PLoS One. 2014 Nov 10;9(11):e112292. doi: 10.1371/journal.pone.0112292 (PMC4226474; doi:10.1371/journal.pone.0112292)
Supplement: Text S1 — CG-MD Simulation. (DOC) [file pone.0112292.s010.doc]

**Supporting Text 1. CG-MD Simulation**

**1.1. CG Simulation Setup**

As described in the main text, we developed a molecular-based model for weakly bound ligand-receptor surface membrane interactions. Weak binding was modeled as a short-ranged Lennard-Jones interaction between a patch of DMPC lipid bilayer and the free end of a fully flexible PEG polymer (with another end fixed in position to emulate the effects of surface confinement as shown in Fig. S6). The Lennard-Jones potential applied between the polymer and lipid patch was calibrated by modulating the magnitude of the binding potential,$\varepsilon_{bind}$, between the end-bead of the DMPC and the end-bead of the lipid to obtain a weakly binding surface interaction that is consistent with pMHC-TCR binding. Three different PEG lengths were simulated, with the system sizes and setup summarized in Table S1.

The Shinoda-DeVane-Klein (SDK) coarse-grained (CG) model was employed to simulate the PEG and DMPC [1,2]. In this model for coarse-graining, each coarse-grained bead represents three heavy atoms and associated hydrogen atoms. Bond stretching ($U_{bond}$) and angle bending ($U_{angle}$) potentials between groups of CG-beads are represented as harmonic functions:

$$U_{bond}(r_{ij})=k(r_{ij}-r_{0})^{2}$$

$$U_{angle}(\theta_{ijk})=k(\theta_{ijk}-\theta_{0})^{2}$$

Here, $k$ is the force constant for the bond or the angle, $r_{ij}$ is the bond length between i-j beads, $\theta_{ijk}$ the angle between i-j-k bead triplets, $r_{0}$ is the equilibrium bond length, and $\theta_{ijk}$ is the equilibrium angle.

Non-bonded interactions are split into contributions from a Lennard-Jones (LJ) potential, and an electrostatic potential including long-range interactions. For the CG-LJ potential ($U_{LJ}^{CG}$) the following functional form is used:

$$U_{LJ}^{CG}(r_{ij})=\left\{\begin{matrix} \frac{3\sqrt{3}}{2}\varepsilon_{ij}\left[\left(\frac{\sigma_{ij}}{r_{ij}}\right)^{12}-\left(\frac{\sigma_{ij}}{r_{ij}}\right)^{4}\right],for\text{ }U_{LJ}^{12-4} \\ \frac{27}{4}\varepsilon_{ij}\left[\left(\frac{\sigma_{ij}}{r_{ij}}\right)^{9}-\left(\frac{\sigma_{ij}}{r_{ij}}\right)^{6}\right],for\text{ }U_{LJ}^{9-6} \end{matrix}\right.$$

As before, $r_{ij}$ represents the distance between i and j beads, while $\sigma_{ij}$ represents the distance when $U_{LJ}^{CG}(r_{ij})=0$, and $\varepsilon_{ij}$ is the well-depth of the CG-LJ potential. One should note that the $U_{LJ}^{12-4}$ form is used for any interactions with water beads, and the $U_{LJ}^{9-6}$ form is used for all other interactions. Again, the depth of the interaction
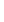
between the tail PEG bead and the head of the DMPC is modified from simple Lenard-Jones type interactions to mimic weak bonding with a new parameter, $\varepsilon_{bind}$, which we set to 2.5 kcal/mol.

For the electrostatic potential ($U_{elec}$), a standard Coulomb interaction is used, which can be written as:

$$U_{elec}(r_{ij})=\frac{q_{i}q_{j}}{4\pi\varepsilon_{0}\varepsilon_{r}}\frac{1}{r_{ij}}$$

In this equation, $q_{i}$ is the charge on the i-th bead, $\varepsilon_{0}$ is the permittivity of free space in rationalized units, $\varepsilon_{r}$ is the relative permittivity of the aqueous medium (80) and implicitly accounts for the electrostatic screening inherent to a polarizable dielectric fluid. The full electrostatic potential including long-range interactions was calculated using the particle-particle particle mesh (PPPM) Ewald summation method as has been recently implemented in HOOMD-blue [3]

**1.2. CG-MD Simulations**

All simulations reported in this work were made using HOOMD-blue graphics processing unit (GPU) accelerated molecular dynamics (MD) simulation engine [4]. The individual simulations were run with 20 independent replicas of approximately 0.5 µs each to improve statistical convergence of the data sets, to obtain a total of 10 µs of date for each set. The setup consisted of roughly 30,000 beads per simulation box as summarized in Table S1. Three-dimensional periodic boundary conditions were used with a 15 Å cutoff distance for all non-bonded interactions.

The simulations were run in the isotropic NPT ensemble with a Nosé-Hoover thermostat [5] and an Anderson barostat [6]. The coupling constants for both the thermostat and barostat were set to 100 ps. The systems were started with a DMPC bilayer that was self-assembled separately in LAMMPS (Large-scale Atomic/Molecular Massively Parallel Simulator) [7] and a random-walk configuration of the PEG polymer. After additional equilibration, separate configurations of the simulation were chosen such that one end of the polymer was found at the prescribed distance above the membrane. For the following simulations, the end was then constrained to this distance above the membrane. To equilibrate these systems, we first ran energy minimization on all particles for 1,000 steps using the Fast Inertial Relaxation Engine (FIRE) algorithm [8]. Following energy minimization, each system was run in the NPT ensemble for 1 ns with velocity rescaling turned on at a frequency of 10 ps^-1^ to equilibrate the system temperature to 303 K and the pressure to 1 atm. After this equilibration period, individual replicated simulations were run for several microseconds each without velocity rescaling. A 10 fs time step was used in all simulations, with a trajectory saving frequency of 1 frame per 0.1 ns. Simulations and data analysis were run on the Longhorn GPU cluster at the Texas Advanced Computing Center (TACC) and on our local Owlsnest GPU cluster at Temple University.

**1.3. Kinetic Data**

In Table S2, we summarize calculated simulation data on the kinetics of the binding interaction. For every frame in the CG-MD simulations, the polymer was characterized as either bound or unbound if the polymer head came within a 1 nm cutoff from the membrane surface (i.e., the model ligand). Using this description, one can qualitatively estimate independently the number of binding events per time (on-rate), and the residence time as the number of consecutive binding events (whose inverse should be proportionally related to the off-rate). In Figure 5, we present our estimates of the on and off rates for the three systems as a function of the polymer Flory radius, scaled against the data for the 4 kDa polymer. This scaling was chosen because of the overlap between the experimental and theoretical conditions. The error bars were calculated using the standard Gaussian error, averaged over all 20 replicas, and then converted into percent error. The error bars for the on rates are indeed small (less than 1% for each point), which can be attributed to the geometric definition of the binding event, which is well sampled over the microsecond timescale for so many replicas. On the other hand, the off rate estimate from the inverse residence time has a large error, which increases as a function of Flory radius. This is likely attributable to the expected increase in the conformational entropy with increasing Flory radius, which would slow the rate of escape of the polymer from the ligand. Our data indicate that a single, multi-microsecond to millisecond length simulation would be needed to properly converge the off-rate error, which is unfortunately outside of the available computational resources. From our model CG-MD data, we find a near linear decay in the on rate as a function of the polymer length. The off rates also decay with increasing Flory radius, however the drop is more consistent with an exponential rather than a linear decay as with the on rates. The potential of mean force between ligand and receptor was ~4 kcal/mol and decreased slightly as PEG length increased. The minimum in the potential of mean force (or the maximum of the radial distribution function) occurred at 0.55 nm and corresponded to an effective physical force of ~40 pN.

**Table S1.** Summary of CG simulation sizes and simulation times.

| Polymer Length | Simulation Time | Number DMPC lipids | System Size | Water | System Size |
| --- | --- | --- | --- | --- | --- |
| 4kDa | 10 µs | 322 | 93 Å x 93 Å x 256Å | 22500 | 26789 |
| 10kDa | 10 µs | 322 | 93 Å x 93 Å x 256Å | 22500 | 26915 |
| 20kDa | 10 µs | 322 | 93 Å x 93 Å x 256Å | 22500 | 27142 |

**Table S2. Summary of calculated simulation parameters, including on-rate, off-rate, and τ_relax_, the relaxation time of the polymer obtained from first order Rouse modes.**

| Polymer Length | On-rate (µs^-1^) | Off±-rate (s^-1^) | τ_relax_ (ns) |
| --- | --- | --- | --- |
| 4kDa | 1.10 x 10^3^ ± 1 x 10^-3^ | 1.39 x 10^9^ ± 2 x 10^8^ | 22.4 |
| 10kDa | 5.37 x 10^2^ ± 5 x 10^-5^ | 0.28 x 10^9^ ± 1 x 10^8^ | 36.2 |
| 20kDa | 1.91 x 10^2^ ± 2 x 10^-5^ | 0.26 x 10^9^ ± 1 x 10^8^ | 201.6 |

**References:**

1. Shinoda W, DeVane R, Klein ML (2007) Multi-property fitting and parameterization of a coarse grained model for aqueous surfactants. Molecular Simulation 33: 27-36.

2. Shinoda W, DeVane R, Klein ML (2010) Zwitterionic lipid assemblies: molecular dynamics studies of monolayers, bilayers, and vesicles using a new coarse grain force field. J Phys Chem B 114: 6836-6849.

3. LeBard DN, Levine BG, Mertmann P, Barr SA, Jusufi A, et al. (2012) Self-assembly of coarse-grained ionic surfactants accelerated by graphics processing units. Soft Matter 8: 2385-2397.

4. Anderson JA, Lorenz CD, Travesset A (2008) General purpose molecular dynamics simulations fully implemented on graphics processing units. J Comput Phys 227: 5342-5359.

5. Nosé S (1984) A unified formulation of the constant temperature molecular dynamics methods. J Chem Phys 81: 511-519.

6. Anderson HC (1980) Molecular dynamics at constant pressure and/ or temperature. J Chem Phys 72: 2384-2393.

7. Plimpton S (1995) Parallel Algorithms for Short-Range Molecular Dynamics. J Comput Phys 117: 1-19.

8. Bitzek E, Koshinen P, Gahler F, Moseler M, Gumbsch P (2006) Structural Relaxation Made Simple. Phys Rev Lett 97: 170201-170204.
