# Supplementary material for: TCR Triggering by pMHC Ligands Tethered on Surfaces via Poly(Ethylene Glycol) Depends on Polymer Length
Source: PLoS One. 2014 Nov 10;9(11):e112292. doi: 10.1371/journal.pone.0112292 (PMC4226474; doi:10.1371/journal.pone.0112292)
Supplement: Text S2 — Calculation of the number of pMHC-PEG-bio bound to each streptavidin on a plastic surface. (DOC) [file pone.0112292.s011.doc]

**Supporting Text 2. Calculation of the number of pMHC-PEG-bio bound to each streptavidin on a plastic surface.**

Assumptions:

- Streptavidin (STP) coated on plastic surfaces has 2 binding sites available for biotin binding.

- All input pMHC-PEG-bio are bound to STP.

Streptavidin (STP) coats well at area density d_stp_ = 6000/um^2^

Average pMHC-PEG-bio density

based on coating concentration d_btn_

Area of T-cell A_cell_ = 50 um^2^

Ratio of pMHC-PEG-bio to STP binding sites r = d_btn_/(2.d_stp_)

Fraction of STP binding 0 pMHC-PEG-bio f_0_ = (1-r)^2^

Fraction of STP binding 1 pMHC-PEG-bio f_1_ = 2.r(1-r)

Fraction of STP binding 2 pMHC-PEG-bio f_2_ = r^2^

(Check: f_0_+f_1_+f_2_ = 1)

Average # of STP’s in contact with one cell n_stp_ = A_cell_d_stp_

Average number of STP’s in contact with one cell with

0 pMHC-PEG-bio bound n(0) = n_stp_f_0_

1 pMHC-PEG-bio bound n(1) = n_stp_f_1_

2 pMHC-PEG-bio bound n(2) = n_stp_f_2_

**Table S3.** Calculated n(1) and n(2) for each of the pMHC-PEG-bio coating concentrations in **Fig. 4**.

|  | **1** | **2** | **3** | **4** | **5** | **6** |
| --- | --- | --- | --- | --- | --- | --- |
| **Coating concentration (pM)** | 7.3E+03 | 1.8E+03 | 4.5E+02 | 1.1E+02 | 2.8E+01 | 7.1E+00 |
| **dbtn** | 4.0E+03 | 1.0E+03 | 2.5E+02 | 6.3E+01 | 1.6E+01 | 3.9E+00 |
| **n(1)** | 1.2E+05 | 3.8E+04 | 1.0E+04 | 2.5E+03 | 6.4E+02 | 1.6E+02 |
| **n(2)** | 2.2E+04 | 1.4E+03 | 8.7E+01 | 5.4E+00 | 3.4E-01 | 2.1E-02 |

|  | **7** | **8** | **9** | **10** | **11** |
| --- | --- | --- | --- | --- | --- |
| **Coating concentration (pM)** | 1.8E+00 | 4.4E-01 | 1.1E-01 | 2.8E-02 | 6.9E-03 |
| **dbtn** | 9.9E-01 | 2.5E-01 | 6.2E-02 | 1.5E-02 | 3.9E-03 |
| **n(1)** | 4.0E+01 | 1.0E+01 | 2.5E+00 | 6.2E-01 | 1.6E-01 |
| **n(2)** | 1.3E-03 | 8.3E-05 | 5.2E-06 | 3.2E-07 | 2.0E-08 |
